# Cell cycle regulation: p53-p21-RB signaling

**DOI:** 10.1038/s41418-022-00988-z

**Published:** 2022-03-31

**Authors:** Kurt Engeland

**Affiliations:** Molecular Oncology, Medical School, University of Leipzig, Semmelweisstrasse 14, 04103 Leipzig, Germany

**Keywords:** Cell biology, Cancer

## Abstract

The retinoblastoma protein RB and the transcription factor p53 are central tumor suppressors. They are often found inactivated in various tumor types. Both proteins play central roles in regulating the cell division cycle. RB forms complexes with the E2F family of transcription factors and downregulates numerous genes. Among the RB-E2F target genes, a large number code for key cell cycle regulators. Their transcriptional repression by the RB-E2F complex is released through phosphorylation of RB, leading to expression of the cell cycle regulators. The release from repression can be prevented by the cyclin-dependent kinase inhibitor p21/CDKN1A. The *CDKN1A* gene is transcriptionally activated by p53. Taken together, these elements constitute the p53-p21-RB signaling pathway. Following activation of p53, for example by viral infection or induction of DNA damage, p21 expression is upregulated. High levels of p21 then result in RB-E2F complex formation and downregulation of a large number of cell cycle genes. Thus, p53-dependent transcriptional repression is indirect. The reduced expression of the many regulators leads to cell cycle arrest. Examination of the p53-p21-RB targets and genes controlled by the related p53-p21-DREAM signaling pathway reveals that there is a large overlap of the two groups. Mechanistically this can be explained by replacing RB-E2F complexes with the DREAM transcriptional repressor complex at E2F sites in target promoters. In contrast to RB-E2F, DREAM can downregulate genes also through CHR transcription factor binding sites. This results in a distinct gene set controlled by p53-p21-DREAM signaling independent of RB-E2F. Furthermore, RB has non-canonical functions without binding to E2F and DNA. Such a role of RB supporting DREAM formation may be exerted by the RB-SKP2-p27-cyclin A/E-CDK2-p130-DREAM link. In the current synopsis, the mechanism of regulation by p53-p21-RB signaling is assessed and the overlap with p53-p21-DREAM signaling is examined.

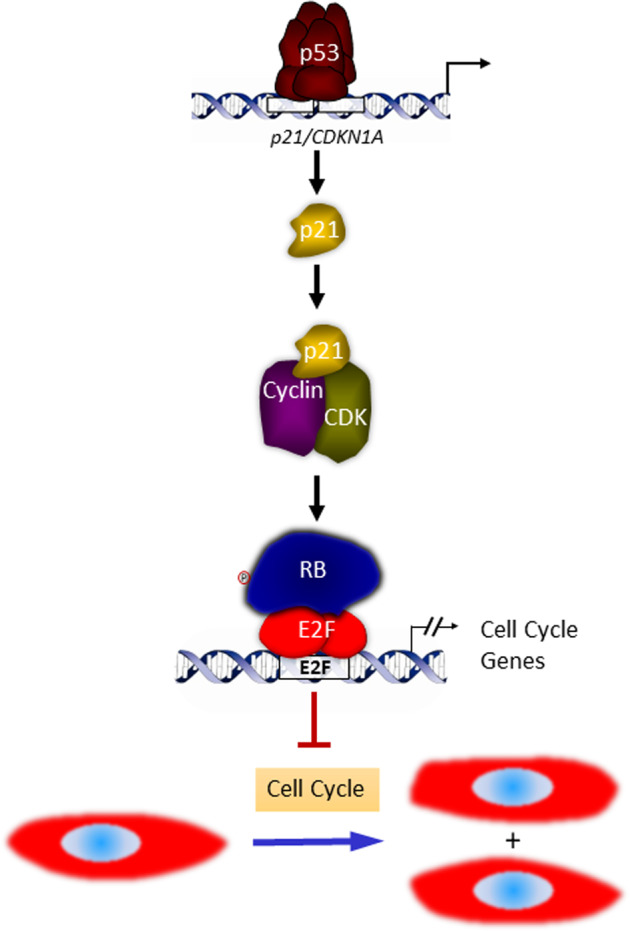

## Facts

- The tumor suppressor p53 can induce cell cycle arrest.
- Induction of p53 leads to transcriptional downregulation of many cell cycle genes.
- The cyclin-dependent kinase inhibitor p21/WAF1/CIP1/CDKN1A is required for downregulation.
- RB-E2F complexes bind to promoters of a large fraction of genes repressed by p53 and p21.
- The resulting p53-p21-RB signaling overlaps substantially with the related p53-p21-DREAM signaling.
- Indirect transcriptional repression by p53 is the result of both signaling pathways.

## Open Questions

- How is overlapping RB-E2F versus DREAM binding to promoters regulated?
- In what non-canonical way can RB affect transcription of DREAM targets without DNA binding by RB-E2F complexes?

### RB and p53 - two important tumor suppressors

The tumor suppressors p53 and RB have prominent roles in blocking cancer development. Their function is connected in several ways. Here, their functional interaction through the cyclin-dependent kinase inhibitor p21/CDKN1A is described. The resulting p53-p21-RB mechanism controls transcription of a large number of genes. Many of these genes are central regulators of the cell division cycle. Thus, loss of p53 or RB function leads to cell cycle dysregulation and malignant proliferation.

### RB function is lost in many tumors

The RB retinoblastoma-associated protein (pRB, *RB1*) represents the first identified tumor suppressor. Loss of RB function is often a central step in cancer development [1, 2]. Inherited mutations in *RB1* were initially identified to predispose for retinoblastoma and osteosarcoma [3]. Later, many tumor types were found to hold inactivated RB arising also from spontaneous alterations, particularly in small-cell lung cancer, glioma, esophageal cancer, and liver tumors [2].

The general importance of RB in tumor suppression is further documented by investigating mice deficient in the Rb protein. Animals heterozygous for *Rb1* mutations develop primarily pituitary and thyroid tumors and various forms of hyperplasia. Interestingly, these animals do not predominantly suffer from retinoblastoma. In addition to tumor development, RB is also important for normal fetal development as homozygous *Rb1* mutations are embryonically lethal in mice [4, 5]. With the advent of large-scale sequencing also of individual tumor samples, data collections as from *The Cancer Genome Atlas - TCGA* (https://www.cancer.gov/tcga) have yielded an unbiased insight into alterations observed in all tumors. According to a data analysis based on 12 tumor types, *RB1* somatic mutations or deletions are detected in a large fraction of colorectal, breast, uterine, ovarian, bladder, and lung cancer [6]. When looking beyond these 12 tumor tissues, it becomes apparent that RB is also found mutated in a large fraction of various cancer types originating from other tissues (*TCGA* database).

Furthermore, RB function can be inactivated by other means than genetic aberrations. In addition to inactivating mutations or deletion of the *RB1* gene, RB function can be abrogated by viral oncoproteins. Oncoproteins from small DNA tumor viruses such as adenovirus E1A protein, simian virus SV40 large tumor antigen, and human papillomavirus HPV E7 protein disrupt the interaction of RB with the E2F transcription factor family [7, 8]. Similarly, large T antigen expressed in cells infected with Merkel cell polyomavirus (MCPyV) contributes to the development of Merkel cell carcinoma (MCC) by forming complexes with RB [9]. Particularly HPV E7 has a substantial impact on the development of human cancer through interference with RB function. All tissues that are susceptible to HPV infection develop tumors. For example, a large fraction of head and neck cancers are thought to be caused by HPV [10]. More importantly, essentially all tumors of the cervix uteri are a consequence of HPV infection [11, 12]. Furthermore, RB can be inactivated through binding of the MDM2 protein, which is often overexpressed in tumors. MDM2 causes proteolytic degradation of RB [13].

Taken together and in contrast to its naming, many tumors of different origins have lost their RB function, either by *RB1* mutation, RB proteolysis, or loss of RB-E2F interaction. These observations suggest a more universal function of RB as a tumor suppressor across most tissues - not only in retinoblastoma.

### Cell cycle control by RB

RB is a central regulator of the cell cycle [1, 14]. Functionally RB represents a transcriptional corepressor. It forms complexes with the E2F family of transcription factors. Importantly, the resulting RB-E2F complexes switch E2F promoter sites from activator to repressor sites [15]. RB binds preferentially E2F1, E2F2, and E2F3, but can also attach to E2F4 and E2F5. These factors form complexes with the DP dimerization partners DP1 or DP2 to form the E2F component of RB-E2F complexes [16–18]. The E2F component of these complexes contacts the DNA via E2F transcription factor binding sites in the gene promoters. RB-E2F complexes downregulate transcription of the genes [19]. Classical RB-E2F target genes often control the cell cycle by contributing to DNA replication and the transition from the G_1_ to S phase. Specifically, genes such as DNA polymerase α (*POLA1*), cyclin A (*CCNA2*), thymidine kinase (*TK1*), dihydrofolate reductase (*DHFR*), cyclin-dependent kinase 1 CDC2/CDK1 (*CDK1*), and minichromosome maintenance complex component 3 and 5 (*MCM3/5*, DNA replication licensing factors) are considered bona fide RB-E2F targets [20, 21].

Curiously, among the originally described RB-E2F targets are many that we later found to rather be controlled by the DREAM protein complex. DREAM is a transcriptional repressor composed of the RB-related proteins p107 (RBL1) or p130 (RBL2), E2F4 or E2F5, DP, and the MuvB core complex [22, 23]. Cyclin A (*CCNA2*) and the cyclin-dependent kinase CDC2 (*CDK1*) together with *CDC25C* were the first genes for which we identified a new transcription factor binding site that we named CHR - cell cycle genes homology region [24]. We later showed that the CHR is essential for binding the MuvB core complex which forms the basis for DREAM [25]. Importantly, in addition to binding CHR elements DREAM can bind also to E2F promoter sites [22]. These results already indicate an overlap or competition between regulatory mechanisms controlled by RB-E2F versus DREAM-MuvB complexes, which will be discussed below.

In addition to repressing transcription by E2F family members, RB exerts functions independent of binding to E2F proteins. Several hundred proteins presumably interact with RB, but it is difficult to assess which of the interactions are functionally relevant [1]. Non-canonical RB functions resulting from these interactions have been suggested, although their significance for cancer development is still elusive [26]. It is not evident which of RB’s functions - transcriptional repression through binding E2F proteins or the interaction with various other factors independent of E2F – are key to its function [1, 26].

Independent of the mechanistic details, it is evident that inactivation of RB causes induction of cell division, defects in cell cycle exit, impaired ability to enter senescence, and compromise of cell-cycle-checkpoint control, particular at G_1_/S transition [1].

### p53 – tumor suppressor and guardian of the genome

Probably the best-known factor relevant for preventing malignancy is p53. The p53 protein is likely also the best studied tumor suppressor. Its main functions are the induction of apoptosis and cell cycle arrest. Furthermore, p53 is involved in DNA repair, control of metabolic pathways, embryo implantation, and driving cells into senescence [27–29]. p53 is inactivated by several viral oncoproteins. This counters that host cells enter apoptosis to limit viral replication and spread [27].

In human cancers, *TP53* is the most commonly mutated gene. An estimate is that on average - across all types - about half of all tumors carry mutations or deletions of this gene. In addition to genetic inactivation, it is assumed that the majority of other tumors have lost p53 function by other mechanisms. For instance, viral oncoproteins or overexpression of MDM2 cause p53 proteolysis [27, 28, 30]. Thus, the widely accepted notion is that most tumors have lost p53 function either by mutation or by compromising the p53 pathway.

p53 is a transcription factor that activates a large number of genes [31, 32]. Prominent targets for its direct transcriptional activation are the apoptosis inducers BAX and PUMA/BBC3 [33, 34]. Importantly, about half of all genes transcriptionally regulated by p53 are repressed. For a long time it was unresolved how p53 initiates downregulation of the many cell cycle regulators it controls, such as cyclin B1 and B2 [35]. It became evident that p53 represses its many target genes indirectly when we performed genome-wide analyses in the context of DREAM-dependent transcriptional repression [36]. Indirect transcriptional repression by p53 requires the cyclin-dependent kinase inhibitor p21/WAF1/CIP1/CDKN1A [23, 37, 38].

### p21 - a CDK inhibitor

The *CDKN1A* gene, coding for the cyclin-dependent kinase inhibitor p21/WAF1/CIP1/CDKN1A, was the first discovered transcriptional target of p53 [39, 40]. Its binding spectrum is wide, as p21 forms complexes with CDK1 (also named CDC2), CDK2, CDK3, CDK4, and CDK6 together with specific cyclins forming complexes with these kinases [41–43]. In contrast to this main function, p21 has also been shown to function as an assembly factor for complexes of D-type cyclins with CDK4/6 at low stoichiometric concentrations [44].

p21 has been shown to mediate p53-induced G_1_ cell cycle arrest [39, 40, 45, 46]. Its induction by p53 and concomitant inhibition of CDKs is considered crucial for p21’s tumor-suppressive role [45]. However, loss of p21 function alone is not sufficient for tumor development. Knockout mice do not develop tumors within seven months after birth and p21 mutations in human cancers are infrequent, with the highest rate of about 10% in bladder carcinomas [46, 47]. Interestingly, in certain tumor cells deficient for p53 function, p21 can still be highly expressed and even display oncogenic properties by deregulation of the DNA replication licensing machinery. Unexpectedly, this subset of tumor cells displays high levels of both p21 and Ki-67 [48]. This oncogenic p21 function has been discussed as an anti-apoptotic property of p21 [49]. Usually, Ki-67 and p21 expression is reciprocal and significant co-expression is not observed. Upon transformation and concomitant loss of p53 function, expression of p21 is low but expression of Ki-67 strongly increases. We have recently shown how p53 inactivation and the resulting loss of p21 induction leads to the increase in Ki-67 expression [50].

Taken together, p21 is strongly induced by p53, resulting in p21’s important impact on cell cycle arrest. However, high p21 levels can arise also independently of p53. Furthermore, the tumor-suppressive role of p21 can - particularly in a mutant p53 context or at low p53 levels and resulting low levels of p21 - convert into an oncogenic function.

### RB-E2F complex formation is controlled by p21

p21 as a tumor suppressor governs RB phosphorylation. Formation of the RB-E2F complexes depends on the phosphorylation status of RB [51]. This protein carries several phosphorylation sites as substrates for cyclin D-CDK4/6, cyclin E-CDK2, cyclin A-CDK2, and cyclin B-CDK1 [18, 52]. Hypophosphorylated RB binds to E2F transcription factor dimers [16–18]. RB-E2F complexes repress transcription of numerous cell cycle genes, many of which are required for G_1_/S transition [17]. The hypophosphorylated state of RB can be switched to hyperphosphorylation through kinase activity of cyclin-CDK complexes. These kinases can be inhibited by p21 [53]. Thus, p21 can stimulate RB-E2F complex formation.

Importantly, loss of p21 function can be compensated to some extent by the CDK4/6 inhibitor drugs palbociclib, abemaciclib, and ribociclib. These drugs are already applied in cancer therapy [54].

In addition, the regulatory circuitry between p21, RB, E2F, and p53 is intertwined in a rather complex way as exemplified by the observation that triple-deletion of *E2f1, E2f2*, and *E2f3* in mouse cells causes an increase in p21 protein levels together with cell cycle arrests at G_1_/S and G_2_/M transitions. Interestingly, deletion of p53 but not inactivation of p21 renders E2F-triple-negative cells susceptible to transformation [55].

### p53-p21-RB signaling

As p21 is a target of p53, it depends on p53 activity. p53 levels are induced by cellular stress, for example following DNA damage or viral infection. Elevated p53 concentrations yield increased p53 transcriptional activity triggering cell cycle arrest and apoptosis [27].

**Fig. 1:**
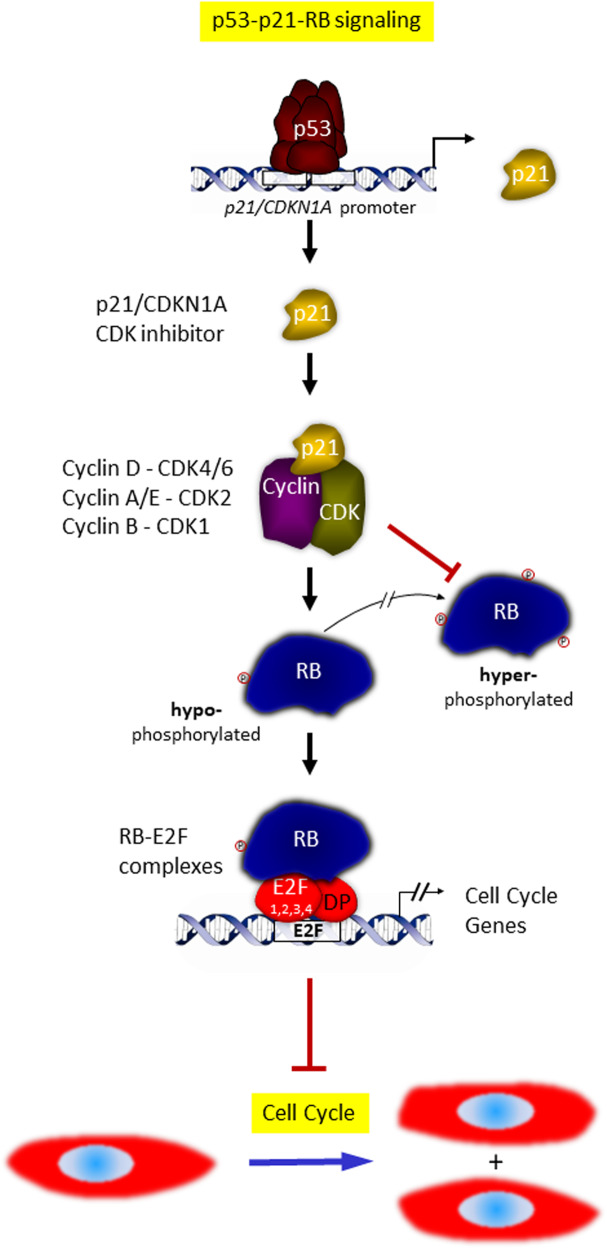
p53-p21-RB signaling. Following p53 activation, transcription of *p21/CDKN1A* is strongly induced as a direct target of p53. The cyclin-dependent kinase inhibitor p21 then blocks activity of several cyclin-CDK complexes. This results in hypophosphorylation of RB, which fosters RB-E2F complex formation and their binding to E2F sites in target promoters. Many target genes are downregulated as a consequence of this mechanism of indirect p53-dependent transcriptional repression. As most repressed genes are involved in cell cycle progression, their downregulation causes cell cycle arrest.

The p21-encoding gene *CDKN1A* is a prominent p53 target (Fig. 1). p53 binds to elements in the p21/*CDNKN1A* promoter and activates its transcription [39]. The p21 protein then inhibits all cyclin-CDK pairs that are involved in hyperphosphorylation of RB.

Hypophosphorylated RB successively forms complexes with E2F transcription factors. RB-E2F complexes downregulate transcription via binding to E2F binding sites in the promoters of target genes.

Taken together, this sequence of events constitutes the p53-p21-RB signaling mechanism (Fig. 1).

### Genes controlled by p53-p21-RB signaling

The p53-p21-RB signaling mechanism significantly contributes to cell cycle regulation and tumor suppression. Therefore, I tried to obtain an overview and identify the genes likely regulated by this mechanism in an unbiased approach. Three criteria were employed to identify p53-p21-RB target genes:

- Genes downregulated upon p53 activation.
- Downregulation lost following p21 inactivation.
- RB and E2F1 binding to promoters.

As the basis for the analyses, a data compilation was used [56]. From this report, 506 genes preselected for RB or E2F binding from a genome-wide dataset were employed to further select genes binding RB and specifically E2F1 [20, 56]. This resulted in a dataset with 488 genes that served as the starting point for further analysis, which is detailed in the legend to Table 1. Using the three selection criteria, the genome-wide analysis yielded 415 genes regulated by p53-p21-RB signaling (Table 1, Supplementary Table S1).

**Table 1:** Genes regulated by p53-p21-RB signaling.

p53-p21-RB targets																	
Gene	RB	DREAM	p53	p21	p53 ChIP	Gene	RB	DREAM	p53	p21	p53 ChIP	Gene	RB	DREAM	p53	p21	p53 ChIP
ABCE1	✓	✓	✓	✓		GPD2	✓	✓	✓	✓		PDS5A	✓		✓	✓	
ACP1	✓	✓	✓	✓		GPR137C	✓	✓	✓	✓		PDS5B	✓	✓	✓	✓	
ACTL6A	✓	✓	✓	✓		GPR180	✓	✓	✓	✓		PHF13	✓		✓	✓	
AFMID	✓	✓	✓	✓		GPRC5B	✓		✓	✓		PHF19	✓	✓	✓	~	
AHCTF1	✓	✓	✓	✓		GRPEL2	✓	✓	✓	✓		PHLPP1	✓		✓	✓	
AK2	✓	✓	✓	✓		GTDC1	✓		✓	~		PIGW	✓	✓	✓	✓	
AMD1	✓	✓	✓	✓		GTPBP2	✓	✓	✓	✓		PIM1	✓	✓	✓	✓	
ANKRD32	✓	✓	✓	✓		GTPBP3	✓	✓	✓	✓		PKP4	✓	✓	✓	✓	
ANLN	✓	✓	✓	✓		H1FX	✓		✓	✓		PLK4	✓	✓	✓	✓	
ANO6	✓		✓	~		H2AFV	✓	✓	✓	✓		PLSCR1	✓	✓	✓	✓	
ANP32E	✓	✓	✓	~		H2AFZ	✓	✓	✓	✓		PNN	✓	✓		✓	
APITD1	✓	✓	✓	✓		HACD2	✓	✓	✓	~		POLA1	✓	✓	✓	✓	
ARL6IP6	✓	✓	✓	~		HAT1	✓	✓	✓	✓		POLA2	✓	✓	✓	✓	
ARRB2	✓	✓	✓	✓		HAUS1	✓	✓	✓	✓		POLD1	✓	✓	✓	~	
ASF1B	✓	✓	✓	✓		HAUS2	✓	✓	✓	✓		POLD2	✓	✓	✓	✓	
ASXL1	✓	✓	✓	✓		HAUS4	✓	✓	✓	✓		POLD3	✓	✓	✓	✓	
ATAD2	✓	✓	✓	✓	✓	HAUS5	✓	✓	✓	✓		POLE	✓	✓	✓	✓	
ATAD3A	✓	✓	✓	✓		HAUS8	✓	✓	✓	✓		POLE2	✓	✓	✓	✓	
ATAD3B	✓	✓	✓	✓		HEATR1	✓	✓	✓	✓		POT1	✓		✓	✓	
ATAD5	✓	✓	✓	✓		HELLS	✓	✓	✓	✓		POU2F1	✓	✓	✓	✓	
ATF2	✓	✓	✓	~		HIST1H2AG	✓	✓		✓	✓	PPAT	✓	✓	✓	✓	
ATF7IP	✓	✓	✓	✓		HIST1H2AH	✓	✓	✓	✓		PPRC1	✓		✓	✓	
AURKA	✓	✓	✓	✓	✓	HIST1H2AK	✓	✓	✓	✓		PRIM1	✓	✓	✓	✓	
AZIN1	✓	✓	✓	✓		HIST1H2AM	✓	✓		✓		PRIM2	✓	✓	✓	✓	
BARD1	✓	✓	✓	✓		HIST1H2BH	✓	✓	✓	n.d.		PRKDC	✓	✓	✓	✓	
BAZ1B	✓	✓	✓	✓		HIST1H2BO	✓	✓	✓	✓		PRR11	✓	✓	✓	✓	
BCS1L	✓	✓	✓	✓		HIST1H3F	✓	✓	✓	✓		PSMC3IP	✓	✓	✓	✓	
BEND3	✓		✓	✓		HIST1H4A	✓	✓	✓	✓		PTGES3	✓	✓	✓	~	
BLM	✓	✓	✓	✓		HIST1H4C	✓	✓	✓	✓		QSER1	✓		✓	✓	
BORA	✓	✓	✓	✓		HIST1H4D	✓	✓	✓	✓		R3HDM1	✓	✓	✓	✓	
BRCA1	✓	✓	✓	✓		HIST1H4I	✓	✓	✓	✓		RAB8A	✓	✓	✓	✓	
BRCA2	✓	✓	✓	✓		HLTF	✓	✓	✓	✓		RAD1	✓	✓	✓	✓	
BRIP1	✓	✓	✓	✓		HMBS	✓	✓	✓	✓		RAD51	✓	✓	✓	✓	
BRIX1	✓	✓	✓	✓		HMGB1	✓	✓	✓	✓		RAD51AP1	✓	✓	✓	✓	
BTG1	✓	✓		✓	✓	HMGB2	✓	✓	✓	✓		RAD54L	✓	✓	✓	✓	
BUB1B	✓	✓	✓	✓		HMGN2	✓	✓	✓	✓		RANBP1	✓	✓	✓	✓	
BUB3	✓	✓	✓	✓		HMGXB4	✓	✓	✓	✓		RBBP4	✓	✓	✓	✓	
C19orf48	✓	✓	✓	✓		HNRNPA0	✓	✓	✓	✓		RBBP7	✓		✓	✓	
C1orf112	✓	✓	✓	✓		HNRNPA1	✓	✓	✓	~		RBL1	✓	✓	✓	✓	
C1orf174	✓	✓	✓	✓		HNRNPA2B1	✓	✓	✓	✓	✓	REXO5	✓	✓	✓	✓	
CAD	✓	✓	✓	✓		HNRNPA3	✓	✓	✓	✓		RFC1	✓	✓	✓	✓	
CAPRIN1	✓		✓	✓		HNRNPAB	✓	✓	✓	✓		RFC2	✓	✓	✓	✓	
CARHSP1	✓	✓	✓	✓		HNRNPD	✓	✓	✓	✓		RFC3	✓	✓	✓	✓	✓
CASP2	✓	✓	✓	✓		HNRNPDL	✓	✓	✓	~		RFC4	✓	✓	✓	✓	
CASP8AP2	✓	✓	✓	✓		HNRNPR	✓	✓	✓	✓		RFC5	✓	✓	✓	✓	
CBX1	✓		✓	✓		HNRNPU	✓	✓	✓	✓		RFWD3	✓	✓	✓	✓	
CBX3	✓	✓	✓	✓	✓	HSP90AA1	✓	✓	✓	✓		RHEB	✓	✓	✓	✓	
CBX5	✓	✓	✓	✓		HSPA8	✓	✓		✓		RIBC2	✓		✓	✓	
CCDC18	✓	✓	✓	✓		HSPD1	✓	✓	✓	✓	✓	RIF1	✓	✓	✓	✓	
CCDC34	✓	✓	✓	✓		HSPE1	✓	✓	✓	✓	✓	RMI1	✓	✓	✓	✓	
CCHCR1	✓	✓	✓	✓		IFT80	✓	✓	✓	✓		RMI2	✓	✓	✓	✓	
CCNE2	✓		✓	✓		IGF2BP1	✓		✓	✓		RMND1	✓	✓	✓	✓	✓
CCT5	✓	✓	✓	✓		ILF3	✓	✓	✓	~		RNASEH2A	✓	✓	✓	✓	
CDC25A	✓	✓	✓	✓		INO80	✓		✓	~		RNF219	✓	✓	✓	✓	
CDC45	✓	✓	✓	✓		INSR	✓			✓		RNF4	✓		✓	✓	
CDC6	✓	✓	✓	✓		INTS7	✓	✓	✓	✓		RNPS1	✓	✓	✓	✓	
CDC7	✓	✓	✓	✓		IPO9	✓		✓	✓		ROCK2	✓		✓	✓	
CDCA7	✓	✓	✓	✓		IQCC	✓		✓	✓		RPA2	✓	✓	✓	✓	
CDCA7L	✓	✓	✓	✓		ITGB3BP	✓	✓	✓	✓	✓	RQCD1	✓	✓	✓	✓	
CDK1	✓	✓	✓	✓		IVNS1ABP	✓		✓	✓		RRM1	✓	✓	✓	✓	✓
CDK2	✓	✓	✓	✓		JADE2	✓			✓		RRM2	✓	✓	✓	✓	
CDK5RAP2	✓	✓	✓	✓		JUND	✓	✓		✓		RSBN1	✓	✓		~	✓
CDKAL1	✓	✓	✓	✓		KAT7	✓	✓	✓	~		RTTN	✓	✓	✓	✓	
CDKN2C	✓	✓	✓	~		KCMF1	✓		✓	✓		S100PBP	✓		✓	✓	
CDKN2D	✓	✓	✓	✓		KDELC2	✓		✓	✓		SAP130	✓		✓	✓	
CDT1	✓	✓	✓	✓		KIAA0101	✓	✓	✓	✓		SENP1	✓	✓	✓	✓	
CENPH	✓	✓	✓	✓		KIF15	✓	✓	✓	✓		SF1	✓		✓	✓	
CENPK	✓	✓	✓	✓		KIF18A	✓	✓	✓	✓		SF3B3	✓	✓	✓	✓	
CENPM	✓	✓	✓	✓		KIF24	✓	✓	✓	✓		SFR1	✓	✓	✓	✓	
CENPN	✓	✓	✓	✓		KIF2A	✓	✓	✓	✓		SIN3A	✓	✓	✓	✓	
CENPQ	✓	✓	✓	✓		KLF11	✓		✓	✓		SIVA1	✓	✓	✓	✓	
CENPU	✓	✓	✓	✓		KMT2A	✓		✓	✓	✓	SKA2	✓	✓	✓	✓	
CEP152	✓	✓	✓	✓		KNOP1	✓	✓	✓	✓		SKP2	✓	✓	✓	✓	
CEP192	✓	✓	✓	✓		KNTC1	✓	✓	✓	✓		SLBP	✓	✓	✓	✓	
CEP295	✓	✓	✓	✓		KPNB1	✓	✓	✓	✓		SLC1A5	✓	✓	✓	✓	✓
CEP55	✓	✓	✓	✓		LARP7	✓	✓		✓		SLC20A1	✓		✓	✓	
CEP57	✓	✓	✓	✓		LCORL	✓	✓	✓	~		SLC25A36	✓	✓		✓	
CEP78	✓	✓	✓	✓		LIN52	✓	✓	✓	✓		SLC25A40	✓	✓	✓	✓	
CHAF1A	✓	✓	✓	✓		LIN54	✓	✓	✓	✓		SLC3A2	✓	✓	✓	✓	
CHAF1B	✓		✓	✓		LIN9	✓	✓	✓	✓		SLMO1	✓		✓	✓	
CHCHD3	✓	✓	✓	✓		LMNB1	✓	✓	✓	✓		SMAD1	✓		✓	~	
CHEK1	✓	✓	✓	✓		LOXL3	✓		✓	✓		SMAD2	✓		✓	✓	
CHRAC1	✓	✓	✓	~		LRR1	✓	✓	✓	✓		SMARCA5	✓	✓	✓	✓	
CISD2	✓	✓	✓	~		LRRC8C	✓		✓	✓		SMC1A	✓	✓	✓	✓	
CLIC4	✓	✓	✓	✓		LRRCC1	✓	✓	✓	✓		SMC3	✓	✓	✓	✓	
CLSPN	✓	✓	✓	✓		LSM3	✓	✓	✓	✓	✓	SMC4	✓	✓	✓	✓	
CLUH	✓		✓	✓		LSM4	✓	✓	✓	✓		SMC5	✓	✓	✓	✓	
CMC2	✓	✓	✓	✓		LSM6	✓		✓	✓		SMPD4	✓	✓	✓	✓	
CNOT1	✓	✓	✓	✓		MAD2L1	✓	✓	✓	✓		SNRPA	✓	✓	✓	✓	
CNP	✓		✓	✓		MAGOHB	✓	✓	✓	✓		SNRPB	✓	✓	✓	✓	
CNTLN	✓		✓	✓		MAP3K5	✓		✓	✓		SNRPD1	✓	✓	✓	✓	✓
COLGALT1	✓		✓	✓		MAT2A	✓	✓		✓		SP1	✓	✓	✓	✓	
CREBZF	✓	✓	✓	✓		MAZ	✓	✓	✓	~		SP4	✓	✓	✓	~	
CTCF	✓	✓	✓	✓		MBOAT1	✓		✓	~		SPATA33	✓		✓	~	
CTDSPL2	✓	✓	✓	✓		MCM10	✓	✓	✓	✓		SPRED1	✓		✓	~	
DBF4	✓	✓	✓	~		MCM2	✓	✓	✓	✓		SRSF1	✓	✓	✓	✓	✓
DCAF16	✓	✓	✓	✓		MCM3	✓	✓	✓	✓		SRSF10	✓	✓	✓	✓	
DCK	✓	✓	✓	✓		MCM4	✓	✓	✓	✓		SRSF2	✓	✓	✓	~	✓
DCLRE1A	✓	✓	✓	✓		MCM5	✓	✓	✓	✓		SRSF7	✓	✓	✓	✓	✓
DCLRE1B	✓	✓	✓	✓	✓	MCM6	✓	✓	✓	~		SSBP2	✓	✓		✓	✓
DCLRE1C	✓	✓		✓		MCM7	✓	✓	✓	✓	✓	SSRP1	✓	✓	✓	✓	
DCPS	✓	✓	✓	~		MCM8	✓	✓	✓	✓		STAG1	✓	✓	✓	~	
DDX11	✓	✓	✓	✓		MCMBP	✓		✓	✓		STIL	✓	✓	✓	✓	
DDX20	✓	✓	✓	✓	✓	MDC1	✓	✓	✓	✓		STMN1	✓	✓	✓	✓	
DDX21	✓	✓	✓	✓		MEIS2	✓	✓	✓	✓		SUPT16H	✓	✓	✓	✓	
DDX46	✓	✓	✓	✓		METTL2B	✓	✓	✓	✓		SUZ12	✓	✓	✓	✓	
DDX55	✓		✓	✓		MIS12	✓	✓	✓	✓		TAF5	✓	✓	✓	✓	
DEK	✓	✓	✓	✓		MMS22L	✓	✓	✓	✓		TCAF1	✓		✓	✓	
DGCR8	✓	✓	✓	✓		MND1	✓	✓	✓	✓		TCF19	✓	✓	✓	~	
DHX15	✓	✓	✓	~		MRPL17	✓	✓	✓	✓		TCP1	✓	✓	✓	✓	
DIAPH1	✓		✓	✓		MSH2	✓	✓	✓	✓		TFAP4	✓	✓	✓	✓	
DLEU1	✓	✓	✓	~		MSH3	✓	✓	✓	✓		TFDP1	✓	✓	✓	✓	
DNA2	✓	✓	✓	✓		MSH5-SAPCD1	✓	✓	✓	~		TICRR	✓	✓	✓	✓	
DNAJC9	✓	✓	✓	✓		MSH6	✓	✓	✓	✓		TIMELESS	✓	✓	✓	✓	
DNMT1	✓	✓	✓	✓		MTBP	✓	✓	✓	✓		TIPIN	✓	✓	✓	✓	
DONSON	✓		✓	✓		MTF2	✓	✓	✓	✓		TK1	✓	✓	✓	✓	
DPYSL2	✓	✓	✓	~		MTHFD1	✓	✓	✓	✓		TLE1	✓		✓	✓	
DSN1	✓	✓	✓	✓		MUTYH	✓	✓	✓	✓		TMEM138	✓	✓	✓	✓	
DTL	✓	✓	✓	✓		MYB	✓		✓	✓		TMEM18	✓	✓	✓	✓	
DUT	✓	✓	✓	✓		MYBL2	✓	✓	✓	~		TMEM194A	✓	✓	✓	✓	
E2F1	✓	✓	✓	~		MYC	✓	✓	✓	✓		TMPO	✓	✓	✓	✓	
E2F2	✓	✓	✓	~		MYO19	✓	✓	✓	✓		TMX1	✓	✓	✓	✓	
E2F7	✓	✓		✓	✓	MZT1	✓	✓	✓	✓		TNPO3	✓	✓	✓	✓	
E2F8	✓	✓	✓	~		NASP	✓	✓	✓	✓		TOE1	✓	✓	✓	✓	
ELP5	✓	✓	✓	✓		NAV2	✓		✓	~		TOPBP1	✓	✓	✓	✓	
EMP2	✓		✓	✓		NCAPD3	✓	✓	✓	✓		TOR1AIP1	✓	✓	✓	✓	✓
ERLIN1	✓		✓	✓		NCAPG	✓	✓	✓	✓		TPR	✓		✓	✓	
ESCO2	✓	✓	✓	✓		NCAPG2	✓	✓	✓	✓		TRA2B	✓	✓	✓	✓	✓
EXO1	✓	✓	✓	✓		NCAPH2	✓	✓	✓	✓		TREX1	✓			✓	
EXOSC2	✓	✓	✓	✓		NCOA5	✓	✓	✓	✓		TRIM37	✓	✓	✓	✓	
EXOSC8	✓	✓	✓	✓		NEDD4	✓		✓	✓		TSR1	✓		✓	✓	
EXOSC9	✓	✓	✓	✓		NF2	✓	✓	✓	✓		TTI1	✓	✓	✓	✓	
EZH2	✓		✓	✓		NFATC2IP	✓	✓	✓	✓		TTLL7	✓			✓	
FAF1	✓	✓	✓	✓		NFIC	✓		✓	✓		TUBG1	✓	✓	✓	✓	
FAM111A	✓	✓	✓	✓		NOC3L	✓	✓	✓	✓		TYRO3	✓		✓	✓	
FAM111B	✓	✓	✓	✓		NOLC1	✓	✓	✓	✓		UBE2R2	✓	✓	✓	✓	
FAM161A	✓	✓	✓	~		NOP58	✓	✓	✓	✓		UBR7	✓	✓	✓	✓	
FAM178A	✓	✓	✓	✓		NPAT	✓	✓	✓	✓		UBTF	✓			✓	
FAM76B	✓	✓	✓	✓		NUCKS1	✓	✓	✓	✓	✓	UCHL5	✓	✓	✓	✓	
FANCC	✓	✓	✓	✓		NUDT21	✓		✓	✓		UCK2	✓		✓	✓	
FANCD2	✓	✓	✓	✓		NUF2	✓	✓	✓	✓	✓	UHRF1	✓	✓	✓	~	
FANCE	✓	✓	✓	✓		NUP107	✓	✓	✓	✓		UNG	✓	✓	✓	✓	
FANCG	✓	✓	✓	✓		NUP155	✓	✓	✓	✓		USP1	✓	✓	✓	✓	
FANCI	✓	✓	✓	✓		NUP188	✓		✓	✓		USP37	✓	✓	✓	✓	
FANCL	✓	✓	✓	✓		NUP205	✓	✓	✓	✓		USP39	✓	✓	✓	✓	
FBXO4	✓	✓	✓	~		NUP50	✓	✓	✓	✓		WDHD1	✓	✓	✓	✓	✓
FBXO5	✓	✓	✓	✓		NUP62	✓	✓	✓	✓		WDR62	✓	✓	✓	~	
FEN1	✓	✓	✓	✓		NUP85	✓	✓	✓	✓		WDR76	✓	✓	✓	✓	
FKBPL	✓	✓	✓	✓		NUP98	✓	✓	✓	✓		WDYHV1	✓	✓	✓	~	
FOXRED1	✓	✓	✓	✓		NUPL1	✓	✓	✓	✓		WEE1	✓	✓	✓	✓	
FUS	✓	✓	✓	✓		NUSAP1	✓	✓	✓	✓		WHSC1	✓	✓	✓	✓	
GABPB2	✓	✓		✓		OARD1	✓	✓		✓		WHSC1L1	✓		✓	✓	
GART	✓	✓	✓	✓		OIP5	✓	✓	✓	✓		WRN	✓	✓	✓	~	
GATAD2A	✓	✓	✓	✓		ORC1	✓	✓	✓	✓		XRCC1	✓	✓	✓	✓	
GGCT	✓	✓	✓	✓		ORC3	✓	✓	✓	✓		XRCC2	✓	✓	✓	✓	
GINS1	✓	✓	✓	✓		PAICS	✓	✓	✓	✓		YARS	✓		✓	✓	
GINS2	✓	✓	✓	✓		PAQR4	✓		✓	~		YEATS4	✓	✓	✓	~	
GINS3	✓	✓	✓	✓		PARP1	✓		✓	✓		ZBTB14	✓	✓	✓	~	
GK	✓			✓		PASK	✓	✓	✓	✓		ZGRF1	✓	✓	✓	✓	
GLI3	✓		✓	✓		PATL1	✓	✓	✓	✓		ZMYND19	✓	✓	✓	✓	
GMNN	✓	✓	✓	✓		PBX3	✓	✓	✓	✓		ZNF367	✓	✓	✓	~	✓
GMPS	✓		✓	✓		PCF11	✓	✓		✓		ZRANB3	✓	✓	✓	✓	
GNB4	✓		✓	✓		PCNA	✓	✓		✓	✓						

RB-dependent regulation. A dataset preselected for RB or E2F targets representing 506 genes was employed (Supplementary Table S9 in [56]). This compilation was further reduced by selecting only genes that had a positive *RB binding score* (✓ in “RB” column) by including RB-E2F targets with chromatin immunoprecipitation (ChIP) binding to promoter regions from one E2F1 and two RB datasets [20, 56]. The *RB binding score* was considered positive if at least 2 out of the 3 scores were positive. The resulting 488 RB-E2F target genes formed the basis for further analysis given in Table 1.

Overlap of RB-E2F with DREAM binding. Genes were considered *DREAM binding score* positive (✓ in “DREAM” column) if 4 out of 9 ChIP datasets for DREAM complex components scored positive [56]). Out of the 488 RB-E2F targets, 410 scored positive for DREAM.

Gene repression dependent on p53. Target genes were selected for downregulation of mRNA levels in various cell systems following p53 overexpression, nutlin-3a, or doxorubicin treatment with a *p53 Expression Score* ≤ −5 (✓ in “p53” column; 465 genes). Genes were scored when at least 5 or more datasets showed downregulation over upregulation of mRNA levels out of 20 datasets examined [56].

Requirement for p21/CDKN1A in p53-induced downregulation. p21 requirement for mRNA downregulation following nutlin-3a or doxorubicin treatment was evaluated using datasets obtained with HCT116 *p21*^*−/−*^ cells comparing treated versus untreated controls (Supplementary Table S4 in [56]). Genes with mRNA regulation below log_2_-fold change ≥0.4 and adj. *p* value ≤0.05 upon nutlin-3a or doxorubicin treatment were scored (✓ in “p21” column, 437 genes). Genes with regulation above this threshold or which were not detected in the datasets were also labeled (∼ or n.d. in “p21” column, respectively; 51 genes in both categories). Nevertheless, all genes regulated above this threshold still displayed a change below log_2_-fold <1.

p53 binding to promoters. Binding of p53 by ChIP in the promoter region 2.5 kb up- or downstream from the transcriptional start site of target genes was assessed (✓ in “p53 ChIP” column; 32 genes from all RB targets, 24 genes from p53-p21-RB targets). Genes were considered positive when 4 out of 15 ChIP datasets scored positive [56].

**Fig. 2:**
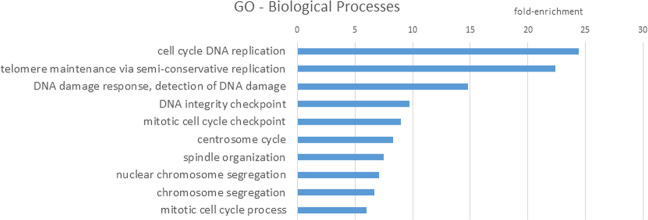
Gene ontology terms biological processes. RB-E2F target genes (488 genes, Table 1) were subjected to GO analysis using DAVID with the criteria: fold-enrichment ≥5; *p* value ≤10^−10^; false discovery rate ≤10^−10^; Fisher’s exact test ≤10^−10^ [83, 84].

When looking at the biological function of this mechanism, cell cycle regulation in general and particularly DNA replication are the processes that are most prominently associated with p53-p21-targets (Fig. 2).

### p53-p21-RB target genes mostly do not bind p53 in their promoters

**Fig. 3:**
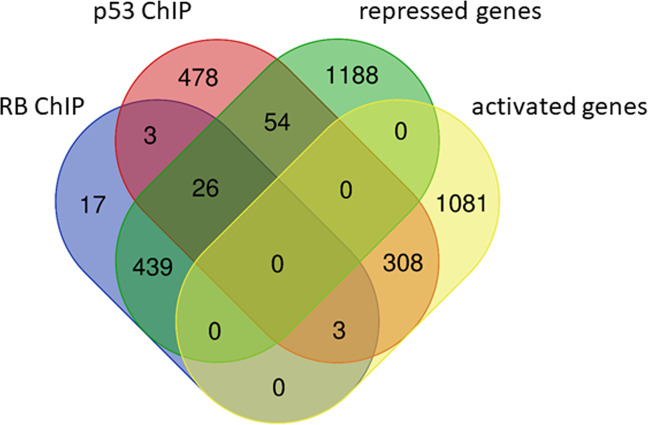
Many genes activated by p53 also bind p53 in their promoters. A large fraction of genes repressed upon p53 induction binds RB-E2F, but essentially all of these genes do not bind p53 in their promoters. Venn diagram depicting p53 and RB binding to promoters of genes regulated by p53. Genes activated (“activated genes”) following p53 induction had a *p53 expression score* of ≥ 5, genes downregulated (“repressed genes”) required a *p53 expression score* of ≤ −5 to be included. Chromatin immunoprecipitation (ChIP) binding of p53 to the promoters of target genes (“p53 ChIP”) had to score ≥5 (Supplementary Table S2 in [56]). The group of RB-E2F targets (“RB ChIP”) are the same 488 genes binding RB and E2F1 as the in “RB” column of Table 1.

**Fig. 4:**
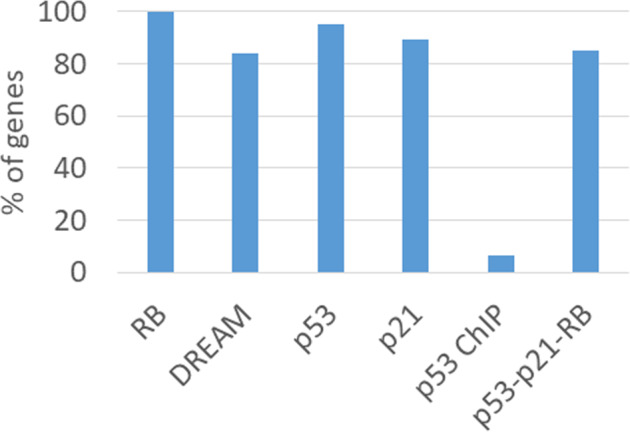
Genes regulated by p53-p21-RB signaling mostly bind DREAM. “RB”: A dataset of 488 genes (100%) selected for RB and E2F1 ChIP binding was used as a basis for the analysis (Table 1). “DREAM”: Genes included if 4 out of 9 ChIP datasets for DREAM complex components scored positive [56]. Out of the 488 RB-E2F targets, 410 out of 488 genes scored positive for DREAM. “p53”: Target genes were selected for downregulation of their mRNA levels following p53 overexpression, nutlin-3a, or doxorubicin treatment (Table 1; 465 out of 488 genes). “p21”: p21 requirement for mRNA downregulation following nutlin-3a or doxorubicin treatment (Table 1; 437 out of 488 genes). “p53 ChIP”: Genes with binding of p53 by ChIP in the promoter region 2.5 kb up- or downstream from the transcriptional start site (Table 1; 32 out of 488 genes). “p53-p21-RB”: Overlap of “RB”, “p53”, and “p21” groups (Table 1; 415 out of 488 genes). Represents p53-p21-RB targets.

In order to evaluate a potential direct regulation by p53, the targets were also checked for p53 binding in their promoter region. Genes activated upon p53 induction often bind p53 in their promoters (Fig. 3). However, in the set of 488 genes binding RB and E2F1 (RB ChIP) just 32 genes are bound by p53 (Fig. 4). Furthermore, for only 24 out of the 415 genes detected as p53-p21-RB targets also p53 chromatin immunoprecipitation (ChIP) binding was detected (Table 1, Fig. 4). A consistent observation with only a few p53-bound genes is made when examining not only RB-E2F targets, but all genes downregulated upon p53 activation (Fig. 3). These results suggest that direct binding of p53 to target promoters does not play a significant role in regulating repressed genes.

### Late cell cycle genes among p53-p21-RB targets

p53 controls checkpoints throughout the cell cycle from G_1_ phase to cytokinesis [27, 57], whereas the classical model associates RB only with cell cycle control during G_1_ and at the G_1_/S transition [14]. In line with this canonical RB model, the analysis provided here finds regulation of genes involved in processes such as nucleotide synthesis and DNA replication (Table 1, Fig. 2). In contrast to the classical RB-dependent regulation, the compilation of p53-p21-RB target genes represents a much larger spectrum of cellular functions. For instance, many cell cycle regulators of later phases were identified, e.g., mitotic spindle assembly checkpoint protein *MAD2L1*, mitotic checkpoint proteins *BUB1B*, and *BUB3* (Table 1, Supplementary Table S1).

Genes regulated by p53-p21-RB signaling are involved in many cell functions particularly associated with cell division. For example, components of the MAP kinase pathway are regulated by the p53-p21-RB axis, e.g., the MAP kinase *MAP3K5/ASK-1*. Another important group of regulated genes are proliferation-related transcription factors such as *SP1, SP4*, Oct-1 transcription factor *POU2F1*, and MYC-associated transcription factor *MAZ*.

Also splicing, as exemplified by helicase *DHX15* and spliceosome component *SNRPA*, as well as regulation of intracellular membrane trafficking with Ras-related protein *RAB8A* as an example are implicated to be controlled by the p53-p21-RB mechanism. The same is true for *CHCHD3*/*MIC19*, a transcription factor and component of the MICOS complex important in the formation of the mitochondria inner membrane. Another subject of regulation is represented by the expression and modification of histones (Table 1, Supplementary Table S1).

Additionally, many genes important for DNA replication and repair are controlled by the p53-p21-RB axis (Fig. 2). Representative examples are *TIMELESS*, the topoisomerase-binding protein *TOPBP1*, the Werner syndrome helicase *WRN*, and several genes from the Fanconi anemia group, e.g., *BRIP1/BACH1/FANCJ* and *FANCC*. For some Fanconi genes regulation through CDE/CHR sites and p53-dependent transcriptional repression has been studied in detail [23, 58].

This examples illustrate just a few of the many functions linked to genes controlled by p53-p21-RB signaling (Table 1, Supplementary Table S1). It is important to appreciate that - when all target genes are coordinately downregulated - the cell cycle will arrest.

### DREAM and RB-E2F - overlap and redundancy

In addition to the many cell cycle regulators that are controlled by the p53-p21-RB mechanism, the most striking observation is that the p53-p21-RB target set broadly overlaps with the list of DREAM targets [23, 36, 38]. From the 415 target genes of the RB pathway, 352 genes or 85% are also identified as p53-p21-DREAM targets (Fig. 4; Table 1; Supplementary Table S1).

This dataset holds various prominent DREAM targets originally described as merely RB-E2F-controlled genes: cyclin-dependent kinase 1 *CDK1* (CDC2) [24, 59], *PLK4* [60], thymidine kinase *TK1* [20, 52], origin recognition complex *ORC1* [61–63], *RAD51* [61], MCM3/5 [62], *CDC25A* [64], DNA polymerases *POLA1*, and *POLD1* [38, 65].

Nevertheless, 63 RB targets appear not to be controlled by DREAM. For instance, important regulators involved in tumor development such as the poly (ADP-ribose) polymerase 1 *PARP1*, the MAP kinase *MAP3K5*, the chromatin-remodeling ATPase *INO80*, the histone methyltransferase *KMT2A*, the E3 ubiquitin-protein ligase *NEDD4*, the Rho-associated protein kinase *ROCK2*, the telomere protection protein *POT1*, the transcriptional regulators *SMAD1*, and *SMAD2* are presumably regulated solely by RB and not by DREAM (Table 1).

The group of genes solely regulated by RB is small compared to the overlap group. The large overlap creates redundancy that allows DREAM to substitute for RB-E2F complexes upon loss of RB function. One example for which this redundancy has been studied is MCM5. RB and p130 can both bind, presumably indirectly, to the same segment of the *MCM5* promoter as detected by ChIP. When either RB or p130 concentration in non-dividing cells is lowered, binding of the other protein is increased [20]. This indicates that RB and p130 indirectly bind to the same sites and compete with or substitute for each other. Likely p130 is complexed in DREAM. This suggests a competition or substitution between RB-E2F and DREAM. Thus, inactivation of one of the complexes may be compensated by the other. However, redundant control by RB-E2F and DREAM as described here is only possible when both complexes are able to bind E2F sites in the target promoters.

### E2F, CDE, CLE, and CHR sites: RB-E2F binding versus multiple-site DREAM binding

**Fig. 5:**
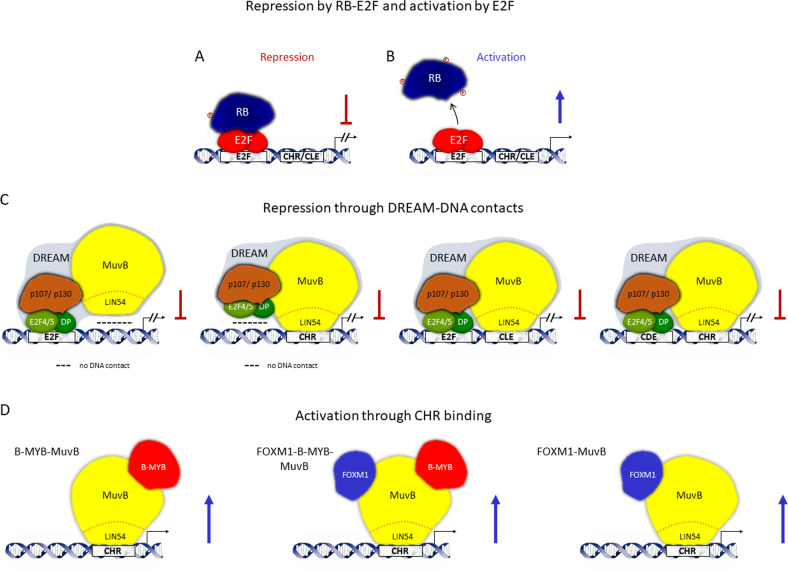
Regulation by RB-E2F complexes is exclusively through E2F promoter sites, but DREAM can contact DNA and repress transcription through four types of promoter sites. B-MYB-FOXM1-MuvB complexes activate genes through CHR sites. **A** Even in the presence of CHR or CLE promoter elements, the RB-E2F complex can only repress transcription through E2F binding elements. **B** Also transcriptional activation, after dissociation of hyperphosphorylated RB from E2F transcription factors, is mediated exclusively through E2F sites. **C** The DREAM repressor complex employs two kinds of subunits for DNA binding. E2F4/5-DP heterodimers bind to E2F and CDE sites. The MuvB core complex component LIN54 contacts CHR and CLE sites. Thus, DREAM can bind DNA through four single or combinations of promoter elements: E2F sites, E2F/CLE, CDE/CHR, or CHR sites. CDE and CLE sites only support DREAM binding. Affinity of DREAM or LIN54-MuvB to CDE and CLE sites alone is not sufficient for productive binding. **D** Following the switch from DREAM to binding of B-MYB and FOXM1 to MuvB, only LIN54 is left as a DNA-binding component. Therefore, gene activation by B-MYB-FOXM1-MuvB complexes is controlled solely through CHR sites.

DREAM can bind to E2F sites but also attaches to sites that RB-E2F does not bind (Fig. 5A, C). Employing their E2F subunits, DREAM and RB-E2F share the ability to bind E2F sites, although the E2F family members in the complexes may vary. However, with the LIN54 subunit, DREAM contains a second protein type that can contact DNA [23, 66]. DREAM binds to cell cycle genes homology region (CHR) sites through its LIN54 subunit (Fig. 5) [65, 66]. This is the basis for regulating an additional set of genes independent of E2F sites and distinct from binding by RB-E2F.

Furthermore, binding of DREAM to CHR or E2F sites can be reinforced by additionally contacting DNA through cell cycle-dependent elements (CDE) or CHR-like elements (CLE), respectively, with a distance of four nucleotides to CHR or E2F sites. Affinity of DREAM to single CDE and CLE sites is not sufficient for regulation [61, 67]. Thus, DREAM can bind promoters through four single or tandem elements: E2F, CHR, CDE/CHR, or E2F/CLE sites (Fig. 5C). In comparison to RB-E2F, DREAM can therefore downregulate a wider spectrum of genes [23, 25, 38, 61]. Gene sets repressed by RB-E2F and DREAM overlap only for genes controlled through E2F sites (Fig. 5A, C).

A similar overlap and division in regulated gene sets is observed when it comes to activation of RB-E2F- and DREAM-controlled genes (Fig. 5B, D) [19, 23, 66]. Following hyperphosphorylation, RB-E2F complexes dissociate and liberate transcriptional activation by the E2F-DP dimer components that bind and activate genes through E2F sites (Fig. 5B). Likewise, p107 or p130 subunits of DREAM become hyperphosphorylated, causing DREAM dissociation. In the absence of DREAM binding and repression through E2F sites, also these target genes can be activated by E2F proteins in later phases of the cell cycle (Fig. 5B). In contrast, genes regulated by CHR or CDE/CHR elements cannot be activated by E2F proteins. These genes become activated through a switch of factors associated with the MuvB core complex. After dissociation of p107/p130, E2F4/5, and DP from DREAM, MuvB successively binds B-MYB and FOXM1 [22, 59]. This switch turns MuvB from a repressor into a transcriptional activator. While the MuvB core complex remains bound to the CHR site, the repressing DREAM complex is changed into activating B-MYB-MuvB, FOXM1-B-MYB-MuvB, and FOXM1-MuvB complexes (Fig. 5D). This CHR-dependent gene set is distinct from the genes repressed through RB-E2F complex. This allows for separate gene set regulation either through CHR sites or dependent on RB-E2F complexes [19, 23, 66].

### Canonical RB-E2F target genes turning out to be DREAM targets

A closer look at transcription factors and selection of their binding sites yields more interesting observations on the regulation of important cell cycle genes. For instance, the oncogenic transcription factor B-MYB (*MYBL2*) had long been recognized as a target for E2F transcription factors trough an E2F binding site in its promoter. When investigating cell cycle-dependent transcription, we confirmed that mutation of the E2F site caused some deregulation of a reporter construct in G_0_ cells. However, we also identified a CHR element downstream of the E2F site. Remarkably, we found that mutation of the CHR led to a more substantial deregulation in G_0_ than mutation of its canonical E2F site [68]. Later we showed that the B-MYB/*MYBL2* CHR site binds DREAM, p53-dependent downregulation of *MYBL2* requires p21, and cell cycle-dependent repression is controlled by DREAM [38, 62, 65]. We had shown by methylation-protection-genomic in vivo footprinting that the *MYBL2* E2F site is occupied in vivo only in G_0_ and G_1_ phase [69]. The nucleotide protection is consistent with both RB-E2F or DREAM binding in resting cells and in G_1_. However, the later loss of protection questions the hypothesis that the E2F site serves as an activator site through binding of uncomplexed E2F after losing RB or DREAM complexing partners in S phase [69]. Our observation was confirmed by detailed chromatin immunoprecipitation experiments showing that E2F4 and p130 bind in G_0_ until mid-G_1_ and are then replaced by E2F1/2/3 until late G_1_. No E2F binding was observed in S phase [70]. Taken together, B-MYB regulation is a good example for an apparently solely RB-E2F-controlled gene that turns out to be also regulated by a CHR element binding DREAM.

Not all classical RB-E2F target genes are exclusively dependent on RB for their transcriptional control. For instance, dihydrofolate reductase *DHFR* appears to be regulated by DREAM and only weakly by RB [23, 56]. Survivin *BIRC5* was reported to be controlled by RB-E2F [71]. However, detailed analysis showed that it is actually regulated by DREAM trough CDE/CHR sites [72].

Another well-documented example is cyclin A (*CCNA2*) [21, 52]. Interestingly, this gene can now be regarded as a classical CHR gene. When we discovered the CHR promoter site, *CCNA2* was among the first CHR genes to be characterized [24]. Later we showed that *CCNA2* is a DREAM target and downregulated by the p53-p21-DREAM pathway [38]. In addition, employing knockout cell models, we found that regulation of *CCNA2* depends on the LIN37 subunit of DREAM but not significantly on RB [63]. These results are consistent with the notion that *CCNA2* downregulation is controlled by CDE/CHR promoter sites binding DREAM. In contrast to the classical view, cyclin A expression appears not to be dependent on RB-E2F complexes but on DREAM/MuvB binding.

In light of the new model combining regulation by the canonical RB-E2F system with control via DREAM/MuvB complexes, the question arises how competing regulation via RB-E2F and DREAM is controlled.

### RB cannot substitute for p107 or p130 in the DREAM complex

One possible overlap could arise from RB as a component of DREAM. However, the DREAM complex is usually found with p107 and p130 as pocket protein components instead of RB [22, 66]. The differential binding is mediated by the LIN52 component of the DREAM complex. LIN52 contacts the RB family pocket proteins through its LxSxExL sequence instead of the usual LxCxE motif. The LxSxExL sequence has a lower affinity to the pocket domains than the standard LxCxE motif. Only phosphorylation of S28 in LIN52 close to the LxSxExL motif by the kinase DYRK1A increases affinity of LIN52 towards p107 and p130 pocket proteins to a similar level as observed for the LxCxE motif. In contrast to p107 and p130, the RB structure lacks residues that could help to stabilize binding with phosphorylated LIN52. These mechanistic observations explain why RB is not found as the pocket protein component in the DREAM complex [73]. Thus, it is not likely that RB indirectly contacts the E2F elements in promoters as a part of DREAM. Instead, it appears that RB binds promoter DNA with E2F proteins only and independent of the MuvB complex (Fig. 5).

### Differential phosphorylation patterns of RB, p107, and p130

Protein phosphorylation is another important factor when searching for distinct properties of RB family members. Differential phosphorylation represents the central mechanism for controlling RB family complex formation. Members of the RB pocket protein family display distinct properties as substrates of kinases. Of the 22 serine and threonine residues phosphorylated in p130, twelve are unique to p130 and ten are conserved in p107. Only three of 22 phosphorylation sites in p130 are found in related peptides of RB. These differences may affect regulation through CDKs and the role of inhibitors such as p21 in modulating CDK activity. Furthermore, the Ser/Thr amino acids in pocket proteins can be phosphorylated also by kinases other than CDKs [74].

One other reason for potentially differing regulation of RB family members could be specificity for RB, p107, or p130 phosphorylation of particular cyclin-CDK combinations. In regard to the control of the p53-p21-RB/DREAM mechanisms, all cyclin-CDK combinations able to phosphorylate RB, p107, or p130 can be inhibited by p21 [41–43].

The generally accepted model explains dissociation of complexes containing RB family proteins through hyperphosphorylation of RB, p107, or p130 by cyclin D-CDK4/6 [52, 75]. Several refinements of this original model have been suggested. For instance, RB is phosphorylated early during the cell cycle by cyclin D-CDK4/6 and later by cyclin E-CDK2 [18, 75]. Cyclin A/E-CDK2 proteins form stable complexes with p107 and p130, but not with RB [52]. Nevertheless, it was reported that cyclin A/E-CDK2 complexes can phosphorylate p130, but not p107 [75]. Also cyclin E-CDK2 complexes contribute to phosphorylation of RB after this pocket protein is initially phosphorylated by cyclin D-CDK4/6 complexes [18, 75]. Additionally, cyclin A-CDK2 and cyclin B-CDK1 keep RB in a hyperphosphorylated state during S phase and later in the cell cycle [18]. Thus, the differential phosphorylation and dephosphorylation of pocket proteins with their impact on complex formation may decide whether RB-E2F or DREAM will form and bind to E2F sites.

### Non-canonical RB function controlling DREAM: the RB-SKP2-p27-cyclin A/E-CDK2-p130-DREAM mechanism

The RB portrait painted so far relates to the refinement of its canonical picture and the comparison with the image we have of DREAM. However, one important question is how RB can exert non-canonical functions independent of its E2F-binding activity [1]. In a recent report, RB was shown to have a substantial role in nuclear organization. RB-induced chromatin dispersion was described that affected expression of 1,627 genes. Consistent with a non-canonical RB function, E2F-regulated genes were not enriched in the chromatin dispersion dataset. Instead, expression of 97 genes related to autophagy were significantly affected by RB-dependent chromatin dispersion [76]. However, DREAM targets were not enriched in the study. Therefore, RB-induced chromatin dispersion does not resolve how RB can control DREAM targets independent of its binding to promoters [76].

There is one recent example for an RB non-canonical regulation of a DREAM target gene, which has been studied in detail. We have recently shown that the *MKI67* gene, which codes for the proliferation marker Ki-67, is bound and regulated by DREAM, B-MYB-MuvB, and FOXM1-MuvB complexes binding through CHR promoter elements [50]. RB-E2F complexes do not bind to the *MKI67* promoter. Nevertheless, *MKI67* expression is partially deregulated when RB is deleted [50]. Hence, Ki-67 serves as an example for a gene mainly regulated by DREAM but with an indirect, non-canonical impact by RB.

**Fig. 6:**
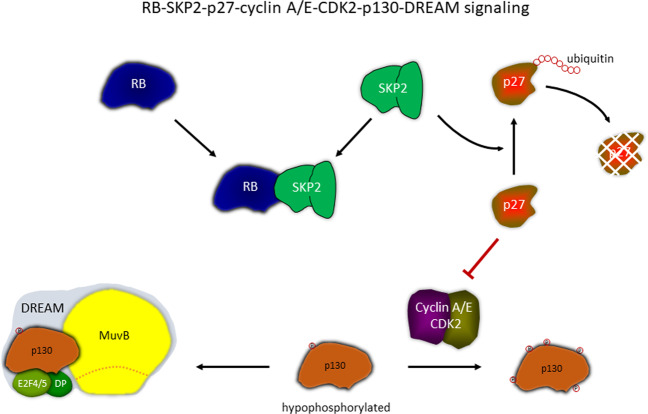
Non-canonical function of RB. Indirect regulation of DREAM formation by RB via the RB-SKP2-p27-cyclin A/E-CDK2-p130-DREAM link. RB can form complexes with SKP2 preventing SKP2 from supporting ubiquitination and degradation of p27 (KIP1, CDKN1B). The CDK inhibitor p27 is then available to block cyclin A/E-CDK2 activity. These cyclin-CDK complexes have a preference for p130 as substrate. Inhibiting p130 phosphorylation yields hypophosphorylated p130, which then enables formation of the DREAM transcriptional repressor.

A possible explanation is provided by a mechanism based on RB-dependent formation of DREAM (Fig. 6). It has been shown that - independent from its canonical function to bind E2F - RB can cause an increase in levels of the CDK inhibitor p27 (KIP1, CDKN1B) [77, 78]. RB binds to SKP2, a component of the SCF complex, and thereby prevents that SKP2 initiates ubiquitination and subsequent degradation of p27 [77]. Furthermore, RB also forms complexes with CDH1 (FZR1), a component of the anaphase-promoting complex/cyclosome (APC/C) complex. CDH1 binding to RB also prevents ubiquitin-dependent p27 degradation [78]. Importantly, p27 can inhibit cyclin E/CDK2 and cyclin A/CDK2 kinase activity. As discussed above, cyclin A/E-CDK2 complexes preferentially phosphorylate the DREAM subunit p130. Thus, p27 stabilization will cause hypophosphorylation of p130 and subsequent DREAM formation (Fig. 6). Taken together, this mechanism describes the RB-SKP2-p27-cyclin A/E-CDK2-p130-DREAM mechanism that enables RB to facilitate DREAM function. This mechanism can explain indirect regulation of DREAM target genes by RB without RB binding to target promoters, as observed for the proliferation marker Ki-67 [50].

### RB, p107, and p130: temporal expression is key

One important difference of RB, p107, and p130 relevant for their function is temporal expression during the cell cycle. Synthesis and degradation affect pocket protein availability and complex formation in the cell cycle [52]. Ubiquitination as a selective modification for differential stability of RB, p107, and p130 is a probable factor, e.g. as recently reported for p130 [79].

Pocket proteins exhibit differential temporal expression patterns. RB is present throughout the cell division cycle. In contrast, concentrations of p107 and p130 vary substantially. p130 is highly expressed in G_0_ and G_1_ phase and expression levels drop in S phase. Inversely, p107 expression is low in G_0_ and beginning of G_1_ with increasing concentration in mid-G_1_ phase and sustained expression into mitosis [52]. These gradually overlapping expression patterns suggest that throughout the cell cycle either p130 or p107 are always present to form DREAM in order to compete for RB-E2F binding at E2F promoter sites.

Taken together, differential phosphorylation of RB, p107, or p130 by varying combinations of cyclin-CDK pairs alone does not provide a distinct mechanism for differential modulation of RB-E2F contrasted with DREAM activities in regard to p53-p21-RB/DREAM signaling, in particular as all the CDKs relevant for all RB family proteins can be inhibited by p21. Rather, temporal expression of RB, p107, and p130 together with differential timing of their phosphorylation and degradation appears to allow for distinct and overlapping functions of RB-E2F versus DREAM.

### p53-dependent transcriptional repression is essentially indirect

RB-E2F and DREAM have in common that they downregulate transcription of target genes following p53 activation. The p53-p21-RB mechanism by definition, as outlined here, represents indirect transcriptional repression by p53. Generally, there has been a long history of discussion about the exact mechanisms of p53 as a repressor of transcription [23, 80]. However, with the advent of genome-wide mRNA expression and ChIP analyses for protein-DNA binding in combination with sophisticated tools in bioinformatics, hypotheses for regulatory mechanisms could be tested in a global manner. We employed several published datasets to analyze all mRNAs differentially expressed upon p53 activation. We found that more mRNAs were downregulated than upregulated [36]. Approximately 2200 mRNAs were induced following p53 activation, while 2700 transcripts were repressed [23].

Models for direct transcriptional repression by p53 require that it is bound to the regulated gene. However, when we searched for p53 binding by ChIP to the genes of the 2700 downregulated transcripts, we found that about 97% of the genes did not significantly bind p53 [23, 36]. These data already suggest an indirect mechanism for p53-dependent repression. Also examining p53 binding to p53-p21-RB targets yields only 24 out of the 415 pathway genes displaying mostly weak p53 binding (Table 1).

More importantly, binding of RB and E2F1 in genome-wide ChIP data is a defining property of genes repressed by p53-p21-RB signaling (Table 1). Thus, transcriptional repression by RB-E2F finally leads to downregulation of RB-E2F target genes following p53 activation (Fig. 1). In summary, transcriptional repression by p53 is largely indirect through RB-E2F and DREAM complexes.

### With a little help from DREAM

#### RB and DREAM pathways – both are required for proper cell cycle control

Generally, tumors that carry mutant RB are also mutated in p53 [1]. This suggests that RB mutation is not sufficient to initiate tumor formation and that some function compensating for RB loss is connected to regulation by p53. An important part of this compensatory p53 function may come from DREAM. Apparently, related functions of RB and DREAM in cell cycle regulation could be key to this compensation. Thus, with the many genes controlled by p53-p21-RB signaling and the large number of targets overlapping with DREAM-dependent regulation, the question arises what impact this mechanism has on cell cycle regulation and whether DREAM can compensate for RB loss.

There are several knockout models that can provide answers. We have employed HCT116 wild-type and knockout cells that were treated with the MDM2 antagonist nutlin-3a and the DNA-damaging agent doxorubicin to increase p53 levels. We observed that wild-type cells can arrest in G_1_ and G_2_/M, whereas p53−/− and p21−/− cells lose their ability to arrest in G_1_ causing accumulation in G_2_/M [63]. Also, we tested mutations in RB and LIN37 [63]. Deletion of the DREAM subunit LIN37 causes loss of DREAM repressor function with a similar phenotype as the combined deletion of p107 and p130 [62].

Interestingly, RB inactivation alone does not significantly change cell cycle distribution in comparison to wild-type cells. Mutation of LIN37 with loss of DREAM function yields a reduction of the G_1_ population upon increased p53 levels, suggesting a significant contribution of DREAM to control at the G_1_/S checkpoint. Importantly, upon combined inactivation of RB and LIN37/DREAM we observed an even more substantial loss of the G_1_ population, causing cells to accumulate in G_2_/M [63]. These results suggest that parallel to the RB pathway also the DREAM pathway has to be inactivated for loss of cell cycle control.

More strikingly, employing an EdU nucleotide incorporation assay, we found that cells continue cycling upon p53 activation only when both RB and LIN37/DREAM are inactivated [63]. Again, these results demonstrate that RB and DREAM synergize in cell cycle regulation and therefore likely in preventing tumor formation.

Overall, our results are consistent with observations from other knockout cell models with deletions of RB, p107, and p130 as single genes or in combinations [81, 82].

When describing signaling pathways relevant for cancer development, a major intention is to identify targets for therapy. In this regard, p53-p21-RB signaling can be rescued by CDK inhibitor drugs when function of p53 or p21 have been lost [54]. In analogy, the same is true for p53-p21-DREAM [23]. Therefore, drugs such as palbociclib, abemaciclib, and ribociclib serve in cancer treatment as they cause cell cycle arrest by reconstituting RB-E2F and DREAM function of both signaling pathways.

In conclusion, p53-p21-RB signaling contributes significantly to cell cycle regulation. RB cooperates with DREAM to cause indirect gene repression and cell cycle arrest following p53 activation.

## Supplementary information

Suppl. Table S1 - p53-p21-RB targets
